# A taxonomy of dignity: a grounded theory study

**DOI:** 10.1186/1472-698X-9-3

**Published:** 2009-02-24

**Authors:** Nora Jacobson

**Affiliations:** 1 Health Systems Research and Consulting Unit, Centre for Addiction and Mental Health, Toronto, Canada; 2 Department of Psychiatry, University of Toronto, Toronto, Canada

## Abstract

**Background:**

This paper has its origins in Jonathan Mann's insight that the experience of dignity may explain the reciprocal relationships between health and human rights. It follows his call for a taxonomy of dignity: "a coherent vocabulary and framework to characterize dignity."

**Methods:**

Grounded theory procedures were use to analyze literature pertaining to dignity and to conduct and analyze 64 semi-structured interviews with persons marginalized by their health or social status, individuals who provide health or social services to these populations, and people working in the field of health and human rights.

**Results:**

The taxonomy presented identifies two main forms of dignity–human dignity and social dignity–and describes several elements of these forms, including the social processes that violate or promote them, the conditions under which such violations and promotions occur, the objects of violation and promotion, and the consequences of dignity violation. Together, these forms and elements point to a theory of dignity as a quality of individuals and collectives that is constituted through interaction and interpretation and structured by conditions pertaining to actors, relationships, settings, and the broader social order.

**Conclusion:**

The taxonomy has several implications for work in health and human rights. It suggests a map to possible points of intervention and provides a language in which to talk about dignity.

## Background

Dignity has long been prominent in the discourses of both health and human rights. In health and healthcare, dignity is featured in many professional practice codes, is a key concept in fields like palliative and long-term care, and also arises in discussions of healthcare service delivery performance and reform [1-5]. Although somewhat controversial in North America, dignity is central to the "new bioethics" that has emerged in Europe over the last decade [6-8]. Dignity and human rights are historically and conceptually coupled in the Universal Declaration of Human Rights [9-11]. Actions taken to respect, protect, and fulfill human rights promote dignity, while those that violate human rights also violate dignity.

As signaled by its title, this paper has its origins in Jonathan Mann's insight that dignity is not only fundamental to health and human rights separately, but may actually serve to explain the link between the two: that is, the relationships between the societal achievement, or failure, of human rights goals and individual and collective health status may be mediated by the experience of dignity [12]. The research upon which this paper is based follows his call for work aimed at developing a taxonomy of dignity: "a coherent vocabulary and framework to characterize dignity" and its violation [13]. Such a taxonomy should describe and classify the forms of dignity, the elements that comprise these forms, and the relationships among the elements, thus expanding understanding of the concept and providing an empirical base from which to develop strategies for enhancing human well-being.

## Methods

Grounded theory is an interpretive research approach with theoretical roots in symbolic interactionism and methodological roots in Chicago School sociology [14,15]. Its practitioners use qualitative methods like interviews and participant observation to gather data, which then are analyzed using the techniques of constant comparison and dimensional analysis [15-17]. The goal of a grounded theory analysis is to derive from the data concepts, conceptual categories, and linkages between categories; the product is a theory of a phenomenon grounded in the lived experience of research participants, rather than an analysis that enlists existing theory to explicate that experience. Unlike some other interpretive methodologies, grounded theory focuses on variation and elaborates complexity, seeking to show how social process makes meaning and how contextual conditions structure social process. These characteristics make grounded theory an excellent methodology to use when investigating concepts like dignity that are simultaneously extremely abstract and strongly rooted in tangible aspects of social life. In addition, because grounded theory "fosters [the integration of] subjective experience with social conditions," it is a valuable tool for social justice research [18].

The methods used for the work reported here were a review and analysis of the literature pertaining to dignity and a series of interviews with individuals for whom dignity is important. The conduct and findings of the literature review have been described at length elsewhere [19]. The data for the analysis described in this paper were drawn from the literature and from 64 interviews conducted with individuals from one or more of three groups: persons marginalized by their health or social status (e.g., people with mental health or addiction diagnoses, homeless individuals); individuals who provide health or social services to these populations (e.g., outreach workers, clergy, healthcare providers); and people working in the arena, broadly defined, of health and human rights (e.g., a street nurse and homeless advocate, a human rights lawyer who specializes in women's reproductive health, a founder of the World Dignity Forum). As is appropriate in grounded theory, all sampling was purposeful. Marginalized individuals were targeted because the extant dignity literature suggested that such individuals are particularly prone to dignity violation. Providers were sampled when the analysis of early data suggested that their perspectives would be important to the developing theory. Health and human rights practitioners were recruited primarily to garner their insights about the political meanings and uses of dignity; these interviews were somewhat less focused on personal experience than were the others. Participants were recruited by flyer and word of mouth (marginalized individuals and service providers) and by invitation (health and human rights practitioners). These methods ensured the salience of the topic for the individuals who agreed to participate. With several exceptions in the groups of providers and health and human rights practitioners, all interviews were conducted with people living and working in Toronto, Ontario, Canada.

The semi-structured interviews, which lasted between 45–90 minutes, used open-ended questions to elicit individuals' detailed accounts of their own experiences of dignity and their understandings of the meaning, impact, and consequences of those experiences. The author and two trained interviewers conducted all interviews; this group met to debrief and discuss the interview questions and responses as the project unfolded. All interviews were audio-recorded and transcribed verbatim. (The research ethics board at the Centre for Addiction and Mental Health in Toronto approved the interview protocol. Participants, who were promised anonymity, provided signed informed consent.) Analysis of the interview data commenced after the first interview and included several steps: coding of transcripts using a coding paradigm from Schatzman's formulation of dimensional analysis, constant comparison of concepts and conditions derived from the data, development of higher order categories to encompass and link these concepts and conditions, and extensive memo writing to track and explore developing ideas [16,17]. As the analysis proceeded, it shaped the interview questions posed to participants (as well as the decision of which participants to recruit), thus allowing a check on the relevance and appropriateness of the interpretation. In addition, at several points a précis of the findings was presented to and feedback invited from individuals and groups who had either participated in or facilitated the research. Analytic work on the taxonomy described here ended once there was a good fit between the data and the categories that make up the taxonomy; that is, the taxonomic elements account for all of the grounded data.

## Results

### Forms of Dignity

A short history of dignity has three main episodes. In the first, man has dignity because he has been made in the image of God. This distinction marks the human species as superior to the rest of creation [20-22]. In the second, dignity resides in social hierarchy and is closely tied to a system of rank. Dignity is both relative–nobles are more dignified than peasants–and absolute–bringing with it a set of duties and privileges tied to office [23,24]. The third episode introduces the Kantian conception of dignity as grounded in the quality of rational agency–the ability of individuals to make moral choices and thus to self-govern [23,25-27]. Dignity is thus an attribute that pertains to humanity as a collective entity, one that attaches only to certain classes of persons, and a characteristic inherent in all individuals.

Contemporary work on dignity, ongoing in disciplines as diverse as theology, philosophy, law, political theory, sociology, medicine, and nursing, is varied and rich, marking dignity as a theoretical and practical concept of enduring fascination. In addition to its foundational roles in health and human rights, dignity also figures prominently in constitutional law, in social justice, and in ethnographic accounts of the lives of poor and oppressed people [28-35]. Despite, or perhaps because of, this ubiquity, the idea of dignity has been criticized for being vague and contradictory [36-38].

The review and analysis of the literature mentioned earlier in this paper led to an understanding that dignity has two complementary but distinct forms: *human dignity *and *social dignity *[19]. Human dignity is the abstract, universal quality of value that belongs to every human being simply by virtue of being human. It is held by the species, by collectives (groups or peoples), and by individuals. It admits of no quantity and cannot be created or destroyed. Social dignity is generated in the interactions between and amongst individuals, collectives, and societies. It may be divided into two types: *dignity-of-self *and *dignity-in-relation*. Dignity-of-self is a quality of self-respect and self-worth that is identified with characteristics like confidence and integrity and a demeanor described as dignified. Dignity-in-relation refers to the ways in which respect and worth are conveyed through individual and collective behavior. It also encompasses the historical sense of dignity as adhering to status or rank. Expectations for what dignity should be and perceptions of when it is present or absent depend upon the mores and traditions of a particular community or society. Because they are socially produced, both types of social dignity are also contingent: they can be measured, violated, or promoted.

Although participants in the interview portions of this study were not asked to define dignity, in speaking of their experiences they often explored their own understandings of its meaning. Their definitions-in-use closely mapped onto the concepts of human and social dignity discerned in the literature. For example, participants described the concept here called human dignity: "dignity is not a commodity...it is inherent in everyone...every person has to be valued being a person"; "it's just something that should be there regardless of who you are or where you are, you know." Dignity-of-self was reflected in remarks like: "dignity is the positive feelings that I have for myself"; "respect, self-esteem, sense of self, self-worth...trust, valuing self, honoring yourself"; "you've got your self-respect, you've got your values, you've got your dignity." And the concept of dignity-in-relation was expressed in comments like: "it's just the way I'm treated and spoken to"; "dignity isn't just how you're treated but also how you treat others"; "respect by others of your own, um, space and your, of who you are"; and "socialism is dignity."

Participants talked about human and social dignity in ways that begin to explain how the two forms of dignity might be related. One participant described dignity-of-self as vulnerable to damage–" [people's dignity is] there, but they may not...they may have been shot down so much that they don't believe that" – but human dignity as enduring – "it's there for everybody, it's just how you access it." A second spoke about human dignity as something that "everybody has inside of them" and social dignity as actions aimed at " [cultivating] the idea of it." Two participants' definitions of dignity, "to be recognized for my real worth as a human being" and "honoring the humanness of the other, or attempting to do that and...being respectful or treating everybody with fairness, and that's what dignity is about," suggested that social dignity (recognition, respect and fair treatment) are responses to or acknowledgments of human dignity (worth as a human being, humanness).

### Elements of Dignity

#### Dignity Encounters

**Figure 1:**
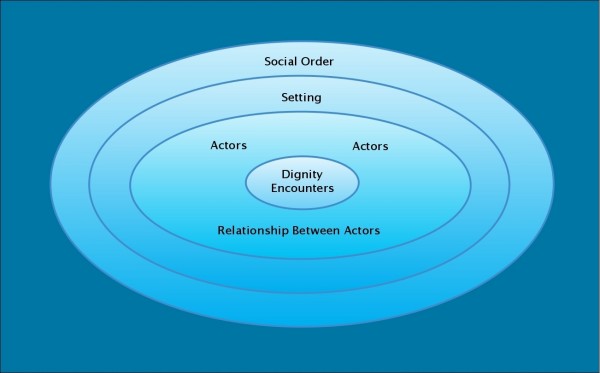
**Conditions of the dignity dimension of an interaction**.

Every human interaction holds the potential to be a dignity encounter–an interaction in which dignity comes to the fore and may be either violated or promoted. Such encounters involve individuals or collectives, actors whose intercourse is composed of layers of markers, gestures, interpretations, and responses. Dignity encounters take place in specific settings, public or private social and physical environments in which actors engage in certain customary patterns of behavior. For example, dignity encounters described by the interview participants unfolded in places like healthcare facilities, family homes, welfare offices, jails, classrooms, coffee shops, and urban sidewalks. These settings, like the actors and their encounters, are embedded in a broader social order. The dignity dimension of an interaction is related to several sets of conditions: the positions of the individual or collective actors; characteristics of the relationship between the actors; features of the setting; and properties of the broader social order in which the setting, actors, and encounter are all situated. Figure 1 is a representation of these conditions.

**Figure 2:**
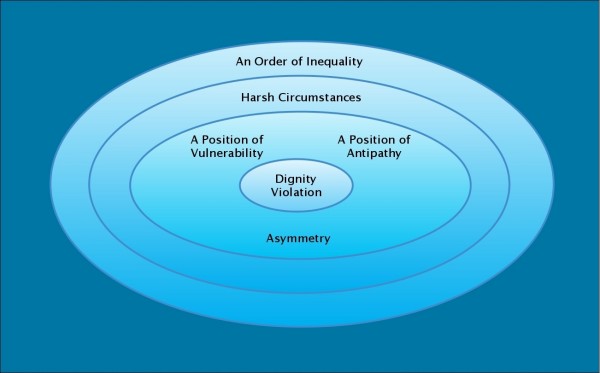
**Portrayal of dignity violation**.

Dignity encounters appear more likely to result in violation when one actor is in a *position of vulnerability*–for example, when the actor is sick, poor, weak, helpless, ashamed, or confused–and the other actor is in a *position of antipathy*–for example, the actor is prejudiced, arrogant, hostile, or impatient. Violation is more common when the relationship between the actors is one of *asymmetry*; that is, when one actor has more power, authority, knowledge, wealth, or strength than the other. Settings characterized by *harsh circumstances *are also more likely to see violation of dignity. Such settings are often described as hierarchical and rigid, full of distraction and stress and urgency, but lacking in resources. Dignity violation is tied to an *order of inequality*, a social order in which inequities like those based in racism or sexism or economic disparity flourish. Dignity violation is portrayed graphically in Figure 2.

**Figure 3:**
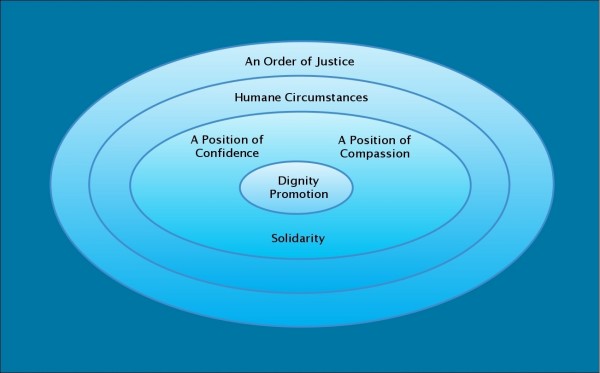
**Portrayal of dignity promotion**.

By contrast, dignity promotion becomes more likely when one actor in an encounter is in a *position of confidence*–has a sense of self-assurance and hope and feels deserving of good things–and the other in a *position of compassion*–is kind, open-minded, honest, and has good intentions. Encounters are more likely to result in dignity promotion when the relationship between the actors is one of *solidarity*; that is, when the relationship is characterized by qualities like reciprocity, rapport, empathy, and trust. Dignity promoting settings are those that feature *humane circumstances*–characteristics like accessibility, transparency, friendliness, beauty, and calm. Finally, dignity promotion is more likely to occur under an *order of justice*, a social order that sees the provision of adequate income and housing, access to education and healthcare, and other societal investment in public goods. Dignity promotion is portrayed graphically in Figure 3.

Participants' accounts suggested these are the ideal conditions for violation and promotion, but that in actuality their dignity encounters often take place in mixed conditions. That is, one may find settings meeting the description of humane circumstances even under broader orders of inequality or relationships characterized by solidarity even when one actor is in a position of vulnerability. Thus, neither dignity violation nor dignity promotion requires the simultaneous presence of all of the conditions described. Conditions at one level can influence conditions at other levels, however. In an order of inequality, the relationship between actors in a given dignity encounter is more likely to be characterized by asymmetry and individual and collective actors more often find themselves in positions of vulnerability. Conditions may be temporally related. Past dignity encounters help to establish the conditions for present and future encounters. An actor whose dignity has been violated in previous encounters is more likely to be in a position of vulnerability in a new encounter, for example. The conditions described should be conceptualized as risk factors for dignity violation or promotion: their presence does not fully determine violation or promotion, but makes such outcomes more likely.

#### Dignity Violation

In a dignity encounter, individual or collective actors engage in a cyclical interaction that involves reading each other's physical and social markers (e.g., age, dress), making gestures that signal the underlying tenor of the interaction (e.g., smiling, avoiding eye contact), interpreting and then responding to these markers and gestures through social processes constituted by word or deed. It is such social processes that violate dignity. Analysis of participants' accounts found that the main social processes involved in their experiences of violation were:

• *Rudeness*: Being gratuitously nasty or showing generalized disrespect.

• *Indifference*: Demonstrating a lack of consideration, heed, or care.

• *Condescension*: "Talking down to" someone or speaking to an adult "like a child."

• *Dismissal*: Ignoring or discounting an actor's knowledge, skills, perceptions, concerns, needs, and feelings.

• *Diminishment*: Making an actor feel smaller or lessened by the form and content of the interaction.

• *Disregard*: Rendering an actor invisible or voiceless.

• *Contempt*: Treating an actor in a way that suggests he or she has no value.

• *Dependence*: Being forced to rely on others for basic needs.

• *Intrusion*: Transgressing an actor's bodily or personal boundaries.

• *Objectification*: Treating an actor like a thing, not a person.

• *Restriction*: Limiting an actor's ability to direct his or her own life.

• *Trickery*: Taunting, lying, or manipulating for material gain or psychological advantage.

• *Grouping*: Seeing an actor not as a unique individual, but only as a member of a collective.

• *Labeling*: Tagging an actor with a descriptive term that carries a connotation of moral deficiency or social inferiority.

• *Vilification*: Making an actor appear threatening or dangerous.

• *Suspicion*: Distrusting or treating an actor as though he or she has committed bad acts.

• *Discrimination*: Treating an actor poorly based on achieved or ascribed status or apparent membership in a low-status group.

• *Exploitation*: Using an actor or viewing him or her only as a means to an end.

• *Exclusion*: Making an actor feel unwelcome in or left out of physical or social settings.

• *Revulsion*: Treating an actor as though he or she is disgusting or tainted.

• *Deprivation*: Lacking absolute or relative access to the necessities of life.

• *Bullying*: Threatening or intimidating an actor.

• *Assault*: Using physical force to damage or demean an actor's body and the spirit.

• *Abjection*: Forcing an actor to humble him or herself by compromising closely held beliefs or by forced association with material or practices considered unclean.

These processes exhibit a number of properties, including temporal properties like duration or frequency; properties of scope, like whether they involve individual or collective actors; properties of visibility, like whether they can be attributed to named actors or whether the actors are anonymous; and properties of intentionality, like whether they are acts of omission or commission. They occur at the micro, meso, and macro levels. For example, a process like deprivation may describe both a child's experience of receiving her siblings' skimpy hand-me-downs and a government's habitual failure to provide adequate health and social services for a segment of its population. Certain processes of violation seem more likely to occur in some settings than in others. Closed environments like jails or family households see some of the more violent processes, like assault. "Micro insults" or processes like rudeness, indifference, and disregard are common in public places like street corners, stores, and restaurants.

For a social process to become a dignity violation requires not only the occurrence of word or deed, but also an act of interpretation. The individual or collective actors involved in the encounter, including any observers who might be implicated, must perceive what transpires and attribute meaning to it. Interpretation itself is a social process structured by the multiple levels of conditions in a given dignity encounter. The actor who exists in a position of vulnerability, for example, may be more likely to read a relatively minor social slight as a dignity violation. In a setting characterized by harsh circumstances, actors may be so worn down from the constant state of arousal that any questioning or refusal of cooperation is interpreted as an attack on dignity.

Thus far, this analysis has presented the social processes of violation as distinct and separate mechanisms. In participants' accounts of violation, however, the social processes of violation tend to cluster. Grouping, labeling, vilification, and discrimination often co-occur, for example, forming social order-level phenomena known as racism, when directed at individuals or groups of color, or stigma, when directed at individuals or groups whose identities have been "spoiled" by a discrediting difference [39,40].

#### Dignity Promotion

The participants in this study were quick to focus on dignity violation in their lives, but had to be prompted to think about times when they experienced an enhancement in their dignity. When they did so, however, it emerged that dignity promotion is a distinct activity–a kind of work, performed by individual and collective agents with the aim of promoting either their own dignity or the dignity of others. This *dignity work *shares with violation its embeddedness in interaction and in conditions pertaining to the actors, the setting, and the social order. It too is constituted by a number of social processes.

The social processes of dignity work described by participants as being conducted by individuals or collectives in order to promote their own dignity are:

• *Contribution*: "Giving something back" to others, as through volunteering.

• *Discipline*: Performing routine activities like cleaning and exercising that are seen as responsible and "normal."

• *Independence*: Being self-sufficient.

• *Accomplishment*: "Doing the job right" or completing an undertaking in a way that meets or exceeds expectations.

• *Authenticity*: "Being myself" or honoring one's own individuality.

• *Creativity*: Making or sharing art.

• *Enrichment*: Making consumption choices that are seen as self-improving.

• *Transcendence*: "Rising above" provocation or temptation.

• *Restraint*: Demonstrating emotional or behavioral reserve.

• *Control*: Taking charge of a situation.

• *Perseverance*: "Just surviving" in difficult circumstances or "making the best of it" after a tragedy or severe disappointment.

• *Preparation*: Steeling oneself, as by reducing expectations, to re-visit settings that have in the past seen violations of dignity.

• *Avoidance*: Steering clear of associates or activities that have in the past led to dignity violation.

• *Concealment*: "Covering up" embarrassing markers or situations.

• *Resistance*: Asserting oneself in the face of threats to dignity.

Participants spoke of the following social processes as dignity work that individuals or collectives perform in order to promote the dignity of others:

• *Recognition*: Acknowledging the humanity of others by paying attention and showing appreciation.

• *Acceptance*: Being non-judgmental of difference.

• *Presence*: Keeping others company in difficult situations.

• *Leveling*: Minimizing asymmetry.

• *Advocacy*: Standing up for, or beside, those who are oppressed.

• *Empowerment*: Working with others to enhance their capacities, capabilities, and competencies.

• *Courtesy*: Demonstrating common respect.

• *Love*: Honoring and esteeming others.

Depending on the nature of the dignity encounter, these micro, meso, and macro level social processes become ways to create dignity where it is lacking, to maintain dignity that may be fragile, to defend dignity that is under threat, or to reclaim dignity that has been lost. For example, a woman hospitalized with a terminal disease defends her dignity by attempting to conceal the markers of her illness, disdaining a hospital gown and putting on full make-up each day. Health and social care providers working with homeless individuals describe how they use processes like presence, advocacy, and empowerment to help create and maintain dignity for their clients. (Such uses of dignity promotion also may be instrumental–helping to keep clients engaged and linked to services.)

#### Objects of Violation and Promotion

When participants spoke about situations in which their dignity was violated or promoted, what is it they understood to have been harmed or enhanced? What are the *objects *of violation or promotion?

Violation and promotion are experienced as causing injury or benefit at two levels: the collective and the individual. At the collective level, participants described how the social processes of violation and promotion, even when enacted in an encounter between individuals, may also offend or benefit *a people *(a group or organization joined by common identification) or *humanity *in general. At the individual level, there are two types of harms and benefits. In the first, people spoke of injuries or benefits to *the self *(identity, self respect, self esteem, individuality, self concept, intelligence, character, self determination, confidence, a sense of one's self as valuable, worthy, or good); violations of or respect for *the body *(bodily integrity); injuries or benefits to *moral agency *(belief, standards, or aesthetics); and offenses to or enhancement of *personhood *(humanness). In the second, participants described how violation and promotion of dignity infringed upon or served *autonomy *(privacy, freedom of choice or movement, "adulthood"); *status *(stature, social standing, role, reputation, visibility); and *citizenship *(the relationship between an actor and the state). These objects correspond to *human dignity *(the people and humanity), *dignity-of-self *(the self, the body, moral agency, and personhood), and *dignity-in-relation *(autonomy, status, and citizenship).

Many of the social processes of violation and promotion are linked to specific objects. For example, at the individual level, aspects of the self are injured by multiple social processes, including rudeness, contempt, bullying, and suspicion, and benefited by others, including authenticity and creativity. The body is violated by intrusion, assault, deprivation, and revulsion. Moral agency is offended by trickery and abjection, but may be enhanced through enrichment and empowerment. Citizenship is degraded by deprivation, exclusion, restriction, and discrimination, and may be advanced through contribution, leveling, or advocacy. Similarly, collective objects like a people may be harmed through processes like vilification, discrimination, exclusion, or exploitation, and greater humanity may be promoted through recognition, acceptance, and courtesy. (Often participants referenced the golden rule, noting that when people treat others as they themselves would wish to be treated the dignity of all is enhanced.)

#### The Consequences of Dignity Violation

Investigators in the social determinants of health have begun to theorize about the pathways through which dignity violation may affect health status [12,41,42]. A taxonomic element that will be important to understanding those pathways, one this paper has not yet discussed, is that of *consequences*. Participants used strong language and vivid examples to speak about the consequences of violation: Violation begins a "dwindling spiral" of damage and loss. When a violation occurs, the individual may experience many emotions, including shock, fear, disbelief, hurt, mortification or embarrassment, discomfort or pain, indignation, frustration, or anger. Initial emotions evolve into a range of longer term experiences of "being wounded": degradation (feeling "worthless," feeling that "you don't deserve anything better," feeling "worn down," feeling "like a failure," feeling "an inch high" or "like a criminal"), humiliation (shame and guilt), anger (resentment and hostility), isolation (no sense of belonging, feeling different from everyone else), insecurity (distrust, dread), disempowerment, and apathy and depression (feeling "like a cork in the water," lack of belief in or valuing anything, feeling suicidal). The individual experiences a series of losses: loss of respect, loss of self worth, ego, sense of self, and soul, loss of status, social standing, and moral standing, loss of confidence and determination. Longer term, the consequences of dignity violation are understood to be social isolation or marginalization, a reluctance to seek help or access resources, passivity or "learned helplessness," a "small" life of constrained choices, chronically poor physical and mental health, and a cycle of victimization and abuse, in which the violated individual turns to violating others. Dignity violation has similar consequences for collectives and whole societies: a group "traumatization," resulting in a lack of balance and the development of a "culture of disrespect" and a subsequent loss of collective dignity ("we all feel less human"). In order to expand our understanding of *how *dignity violation affects individual and collective health status, it will be important for future research to describe and classify these consequences and to link them to specific conditions, social processes, and objects.

## Discussion

The taxonomy of dignity presented in this paper has identified two main forms of dignity–human dignity and social dignity–and has described and classified several elements of these forms, including the social processes that violate or promote them, the conditions under which such violations and promotions occur, the objects of violation and promotion, and the consequences of dignity violation. The companion grounded theory of dignity to emerge from this research sets the static components of the taxonomy in motion, showing how dignity is constituted through social processes of interaction and interpretation that are structured by conditions pertaining to the actors involved, their relationships, the settings, and the broader social order.

Several limitations of this taxonomy should be noted. In presenting the taxonomy in an abstract and generic form, context is lost and, with it, the active interplay among specific social processes and conditions. A more nuanced, and animated, representation would require thick description of particular dignity encounters situated in time and place. Through such close examination, the explanatory depth of the theory would be enhanced, although some of its breadth would be lost.

The taxonomy may or may not be exhaustive and universal in its forms and elements. As noted, there is a long history of defining and explicating dignity in disciplines like philosophy and law. The empirically-based taxonomy described here shows some concordance with the technical meanings of dignity derived through analysis and argument in those fields, but also some divergence. It will be important for future work to subject the taxonomy to interdisciplinary and cross-cultural comparison, allowing for the addition or modification of conditions, social processes, and objects. In some of its emphases, the taxonomy certainly reflects particularities of the environment in which it was developed. For example, the notion of citizenship as an object of violation was very strong in these data, which may be a reflection of specific historical circumstances in Toronto: the city is a major center for arriving immigrants and still feels the effects of a neo-liberal provincial government that, after being elected in the mid-1990s, embarked on a series of budget cuts that left lasting holes in the social safety net and a lasting sense of betrayal among the populace.

## Conclusion

This taxonomy has several implications for work in health and human rights. It serves as a reminder that dignity pertains to both individuals and collective entities and that there is a connection between the two; that is, threats to the dignity of one threaten the dignity of all, and vice versa. Thus, one might expect to see individual and collective health impacts resulting from injuries to dignity that are both very broad and very narrow in scope. The mechanisms of dignity violation described as part of the taxonomy help to elucidate the meso and micro processes embedded in the macro processes of "structural violence" that have been the focus of much attention in the health and human rights movement [43]. Such elucidation can help to explain the connections between societal conditions and health status in individuals and communities.

In addition to its focus on the health impact of human rights violation, the health and human rights approach is concerned with the effects of health policy and health services on human rights [13]. Analyses that look at dignity in healthcare can use this taxonomy to shed light on the nature and consequences of dignity violation in these settings and eventually may provide knowledge that can be used to develop "dignity screens" for health policy development, health program planning, and health services delivery.

Mann's original call did not focus on dignity promotion. However, this study, along with other empirical research, has made it clear that individuals and collectives do act to create, maintain, defend, and reclaim their own dignity and that of others, even in the most difficult situations [1,32,44]. An understanding of dignity work can be extremely useful to advocates and practitioners in health and human rights–who are themselves engaged in a kind of dignity work–allowing them to harness the social processes and conditions of dignity promotion in order to improve health and promote the attainment of human rights goals. This taxonomy can be of value to them in providing both a map to possible points of intervention and a language in which to talk about dignity.

## Competing interests

The author declares that she has no competing interests.

## Authors' contributions

NJ developed the study design, collected and analyzed the data, and wrote the paper. She will act as guarantor for the paper.

## Pre-publication history

The pre-publication history for this paper can be accessed here:

http://www.biomedcentral.com/1472-698X/9/3/prepub
